# Altering crystal growth and annealing in ice-templated scaffolds

**DOI:** 10.1007/s10853-015-9343-z

**Published:** 2015-08-25

**Authors:** K. M. Pawelec, A. Husmann, S. M. Best, R. E. Cameron

**Affiliations:** Department of Materials Science and Metallurgy, Cambridge Centre for Medical Materials, University of Cambridge, Cambridge, CB3 0FS UK

## Abstract

The potential applications of ice-templating porous materials are constantly expanding, especially as scaffolds for tissue engineering. Ice-templating, a process utilizing ice nucleation and growth within an aqueous solution, consists of a cooling stage (before ice nucleation) and a freezing stage (during ice formation). While heat release during cooling can change scaffold isotropy, the freezing stage, where ice crystals grow and anneal, determines the final size of scaffold features. To investigate the path of heat flow within collagen slurries during solidification, a series of ice-templating molds were designed with varying the contact area with the heat sink, in the form of the freeze drier shelf. Contact with the heat sink was found to be critical in determining the efficiency of the release of latent heat within the perspex molds. Isotropic collagen scaffolds were produced with pores which ranged from 90 μm up to 180 μm as the contact area decreased. In addition, low-temperature ice annealing was observed within the structures. After 20 h at −30 °C, conditions which mimic storage prior to lyophilization, scaffold architecture was observed to coarsen significantly. In future, ice-templating molds should consider not only heat conduction during the cooling phase of solidification, but the effects of heat flow during ice growth and annealing.

## Introduction

The process of ice-templating is applicable for many material systems, such as polymers and ceramics. This versatility has allowed ice-templating to be used for applications as diverse as continuous flow catalysts, and even tissue engineering scaffolds [1–3]. During ice-templating techniques, three-dimensional scaffold structures are created through the solidification of a water-based suspension. Within the suspension, ice first nucleates, then grows, rejecting the solute to the volume between ice crystals. Once the ice template is formed, it is removed via sublimation in the drying stage, leaving behind a porous structure which is the inverse of the ice template. In order to tailor the scaffold structure, the growth of the ice template must be controlled, which is, in turn, influenced by the two phases of crystallization: ice nucleation and growth. The thermal behavior of the slurry can thus be separated into two regimes: the “cooling” phase occurring before ice nucleation, and the “freezing” phase which follows nucleation.

During many processes, a tight control over the final product architecture is required. Within the field of regenerative medicine, features such as the pore size of tissue engineering scaffolds, have been shown to play a critical role in cell adhesion and proliferation [4, 5]. The scaffolds produced via ice-templating have already been used to maintain cell phenotype in long-term culture, to create more effective drug delivery devices, and even in the study whole-tissue morphogenesis [6–8].

Most of the research on controlling architecture has focused on the cooling phase of the ice-templating process, which influences the anisotropy of the final structure [9]. If regions of a collagen slurry can nucleate before the entire slurry is below the equilibrium freezing temperature (0 °C), anisotropic structures result [9, 10]. Therefore, heat flow within the slurry is vital in determining the scaffold isotropy, with both mold design and filling volume playing a critical role by controlling the path of heat flow within the slurry [9, 10].

However, scaffold architecture can also be influenced by the freezing phase of solidification. The final pore size reflects the growth and annealing of ice within the slurry, especially while the slurry is around the equilibrium temperature, when it is most prone to rearrangement of the crystal structure [11, 12]. Even at temperatures lower than −50 °C, ice annealing has been shown to occur in sucrose solutions, although at much slower rates [13]. While it is known that crystal growth and annealing processes underlie scaffold structure, little research has focused on how to control the growth of ice during ice-templating techniques beyond altering the freezing protocol.

The goal of the current study was to understand how the freezing phase of solidification influences the final structure of collagen scaffolds, independently of the cooling phase. Given the importance of heat flow, it was hypothesized that the filling volume and design of the ice-templating mold would play a major role in establishing the thermal link between the heat sink and the slurry during solidification. Ice-templating molds with varying contact area with the heat sink were designed and tested with different filling volumes. The final scaffold structures obtained were characterized and linked to the fundamental aspects of ice growth underlying ice-templated structures. To further investigate the influence of low-temperature ice annealing, scaffold structure was examined after a thermal hold below −20 °C, simulating storage conditions. The insight gained can serve as a guideline for the design of ice-templating molds and the long-term storage of frozen structures.

## Materials and methods

### Scaffold production

Collagen suspensions of 1 wt% were prepared from bovine Achilles tendon, type I collagen (Sigma Aldrich), hydrated in 0.05 M acetic acid. Slurries were homogenized for 30 min at 13500 rpm in an ice water bath (VDI 25, VWR International Ltd, UK), and centrifuged (Hermle Z300) for 5 min at 2500 rpm. Prepared suspensions were poured into freeze drying molds to a filling height of either 5 or 15 mm, as it has been shown that as the distance from the heat sink changes, the heat flow through a slurry is also affected [9]. The freezing behavior, was thus expected to be different for 5 mm filling (small volumes) and 15 mm filling (large volumes).

The slurry was cooled at a rate of 0.9 °C min^−1^, and held for 90 min at −30 °C. Frozen slurries were then lyophilized using a Virtis freeze drier (SP Industries, USA) at 0 °C for 20 h under a vacuum of less than 100 mTorr. As a further test of the effects of freezing time on scaffold structure, slurry was kept at −30 °C for 20 h, without changing any other portion of the freezing protocol.

**Fig. 1:**
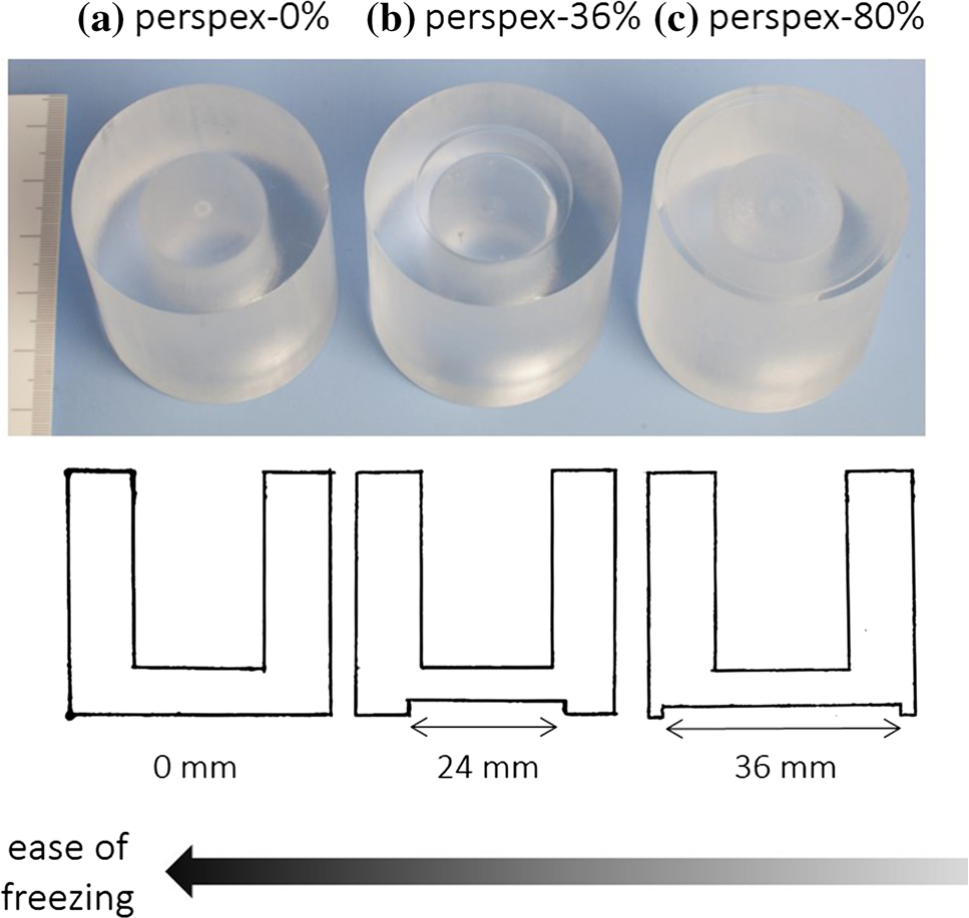
The molds used during the study had different contact areas with the sink (the freeze drier shelf) by varying the size of the air gap at the base ** a** perspex-0 %, ** b** perspex-36 %, and ** c** perspex-80 %. Schematics of each mold are presented below in the pictures

Three molds were designed with a low surface area to volume ratio, in which the contact with the heat sink varied. Each had an inside diameter of 20 mm and height of 30 mm, and walls of 10-mm-thick perspex. The contact area at the base of the molds was varied by adding a counterbore. Mold contact area varied from 1260, 804, to 239 mm^2^. This corresponded to a 0, 36, and 80 % air gap at the base, respectively, and the molds were called “perspex-0 %”, “perspex-36 %”, and “perspex-80 %”, (Fig. 1).

### Pore size analysis

Pore size was determined at the top and base of each scaffold, near the thermocouple. In preparation for pore size analysis, X-ray micro-computed tomography (μCT), Skyscan 1072, scans were taken of 3 mm scaffold sections at a magnification of ×75, (25 kV, 132 μA, 7.5 s exposure time). Reconstructions were performed with the software program NRecon (Skyscan), with a resolution of less than 5 μm. Scans were compared to micrographs of the scaffolds taken with scanning electron microscopy to verify that all of the features were captured. The average pore size was determined using the line-intercept method [14]. Twelve sections were analyzed from each scaffold, using three lines per image.

### Thermal characterization

**Fig. 2:**
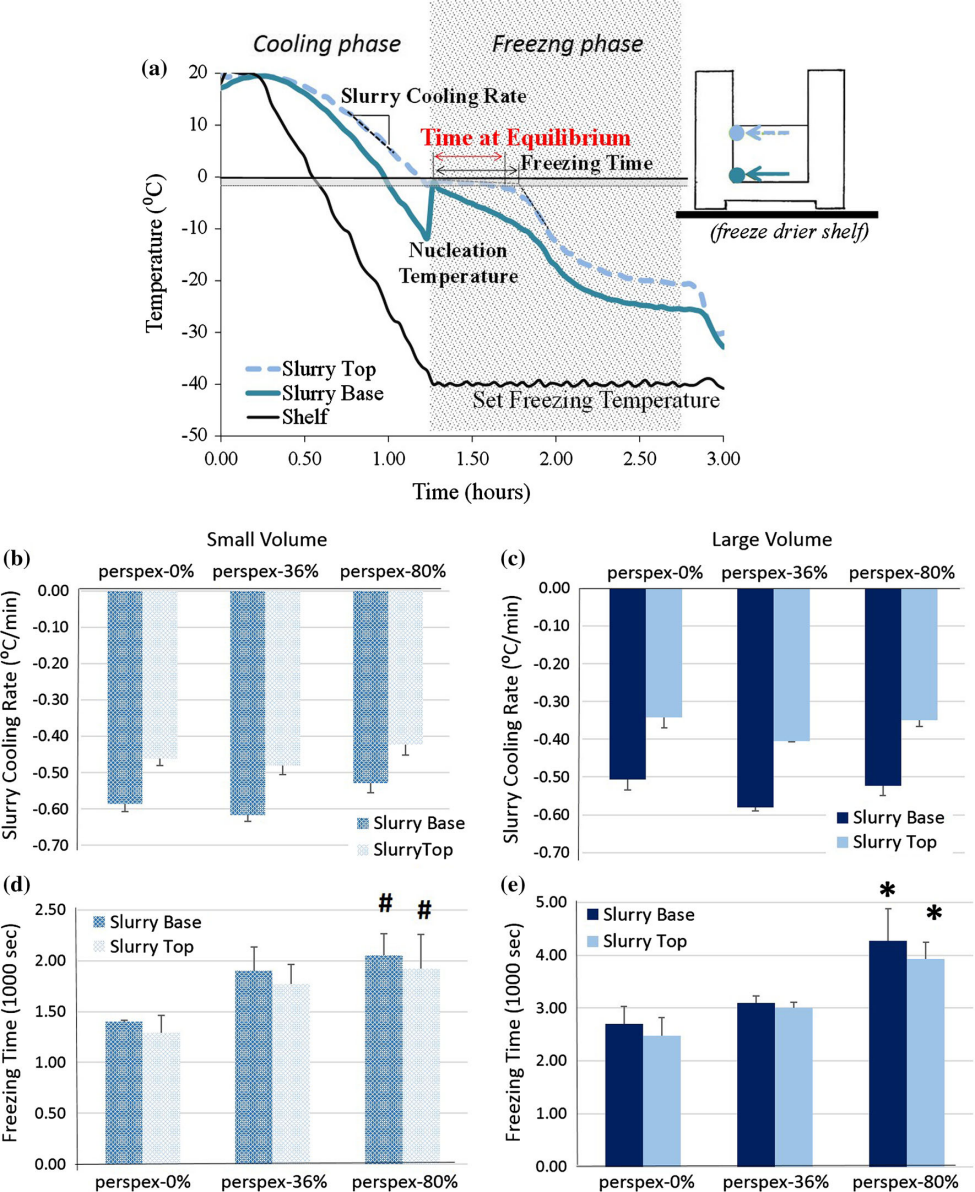
The thermal parameters quantified from thermal profiles were all affected by mold design. **a** Schematic of a thermal profile of a slurry during ice-templating with the cooling and freezing phases marked. The slurry cooling rate was not affected by the air gap with **b** small or **c** large filling volumes. The freezing time varied significantly with the contact with the heat sink with both the **d** small and **e** large filling heights. *Significantly different from all other molds. ^#^Significantly different from perspex-0 % (*p* < 0.05)

During freezing, thermocouples, wired into the Virtis freeze drier (SP Industries, USA), were held in place at base and top of the slurry at the mold wall, as pictured in Fig. 2a; this corresponded to a height from the mold bottom of 0 and 5 or 15 mm for small and large filling volumes. Measurements were recorded every 10 s using a computer program written in LabVIEW. From the thermal curves, the cooling rate of the slurry and freezing time were quantified. Freezing was considered to be initiated when the nucleation temperature was reached, or, if no nucleation occurred, freezing was considered to be initiated when the thermal curve crossed the equilibrium freezing temperature, 0 °C. The end of freezing was defined as the extrapolated point at which the initial slope of the thermal line changed. Freezing time was measured between these two points. The time at equilibrium was defined previously, and refers to the time in which the slurry spends between 0 and −1.5 °C when ice remodeling is most active [11]. Figure 2a illustrates the parameters measured.

### Statistics

For each mold and filling volume, slurry freezing from 3 independent freezing cycles was compared. A confidence interval of 95 % was used throughout. The cooling rate and freezing time were compared via a non-parametric, two-tailed Mann–Whitney test. All other variables were analyzed via linear regression. The results in the figures are presented with standard deviations.

## Results

### Phases of solidification

During the cooling phase of solidification, there was no effect of contact with the heat sink. The slurry cooling rate was only dependent on the position within the slurry. The three perspex molds had cooling rates which were significantly lower at the top of the slurry than at the base. The cooling rates were around −0.53 and −0.37 °C min^−1^ at the base and top of the slurry, respectively. Even as the filling volume increased, the trend remained the same.

In contrast to the cooling phase, mold design changed the freezing behavior of the slurry significantly. The freezing time of the scaffolds was dependent on the contact with the heat sink, demonstrating that this was an important route for the escape of latent heat from the slurry. The longest freezing times, regardless of filling volume, occurred in perspex-80 %; with a large volume, the freezing time was 71.14 ± 10 min, (Fig. 2). Increasing the filling volume also increased the freezing time, which is consistent with previous literature that heat flow can be affected by filling volume [9]. Even when no air gap was present, perspex-0 %, the freezing time increased from 23.31 ± 3 to 45.00 ± 5 min as the filling volume went from small to large.

### Pore size homogeneity

**Fig. 3:**
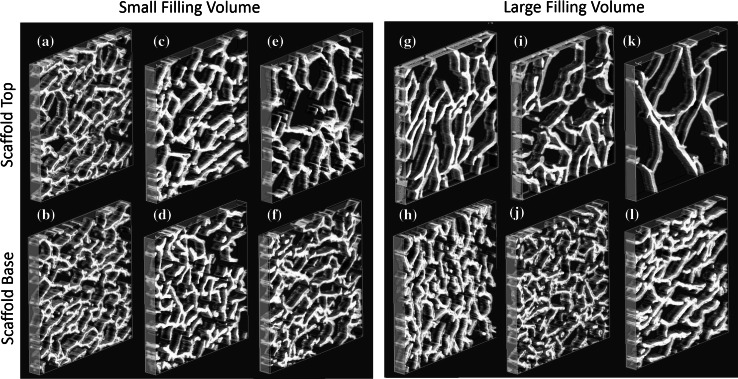
Scaffold pore size, imaged via µCT, was affected by the filling height and mold design. With small volumes there were few differences between ** a**, ** b** perspex-0 %, ** c**, ** d** perspex-36 %, ** e**, ** f** perspex-80 %. When the filling volume became large, the contact with the heat sink altered the pore size ** g**, ** h** perspex-0 %, ** i**, ** j** perspex-36 %, ** k**, ** l** perspex-80 %. All sections are 1 × 1 mm

**Fig. 4:**
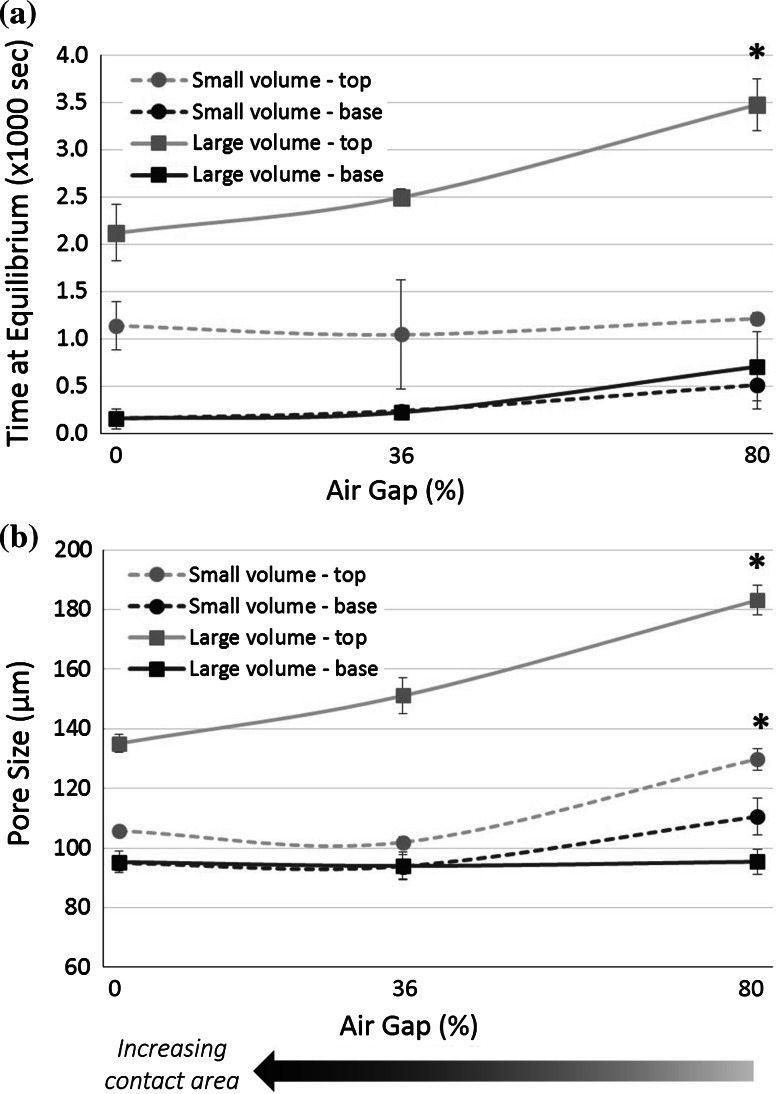
As the percent air gap in the mold varied, the **a** time at equilibrium and the **b** corresponding pore size in the scaffolds also changed. *Significant increase with air gap. (*p* < 0.05)

Scaffolds with isotropic pores resulted in all of the molds and filling heights tested, (Fig. 3). The pore size depended on both the filling volume and the ice-templating mold. It has been previously postulated that the pore size is linked to the time at equilibrium of the slurry, and similar trends were observed in the current study, (Fig. 4) [11].

With small filling volume, there was little change in the pore size due to mold design. However, with large filling volumes, the pore size at the scaffold top increased significantly as the size of the air gap increased, from 135 ± 3 μm for perspex-0 % to 183 ± 5 μm for perspex-80 %. The strong relationship between the pore size and the percentage of air gap is illustrated in Fig. 4. With larger filling, the mean pore size was significantly larger at the top of the scaffold than at the base. At the scaffold base, the pore size did not vary significantly despite changes in mold design, but remained between 92 and 95 μm.

**Fig. 5:**
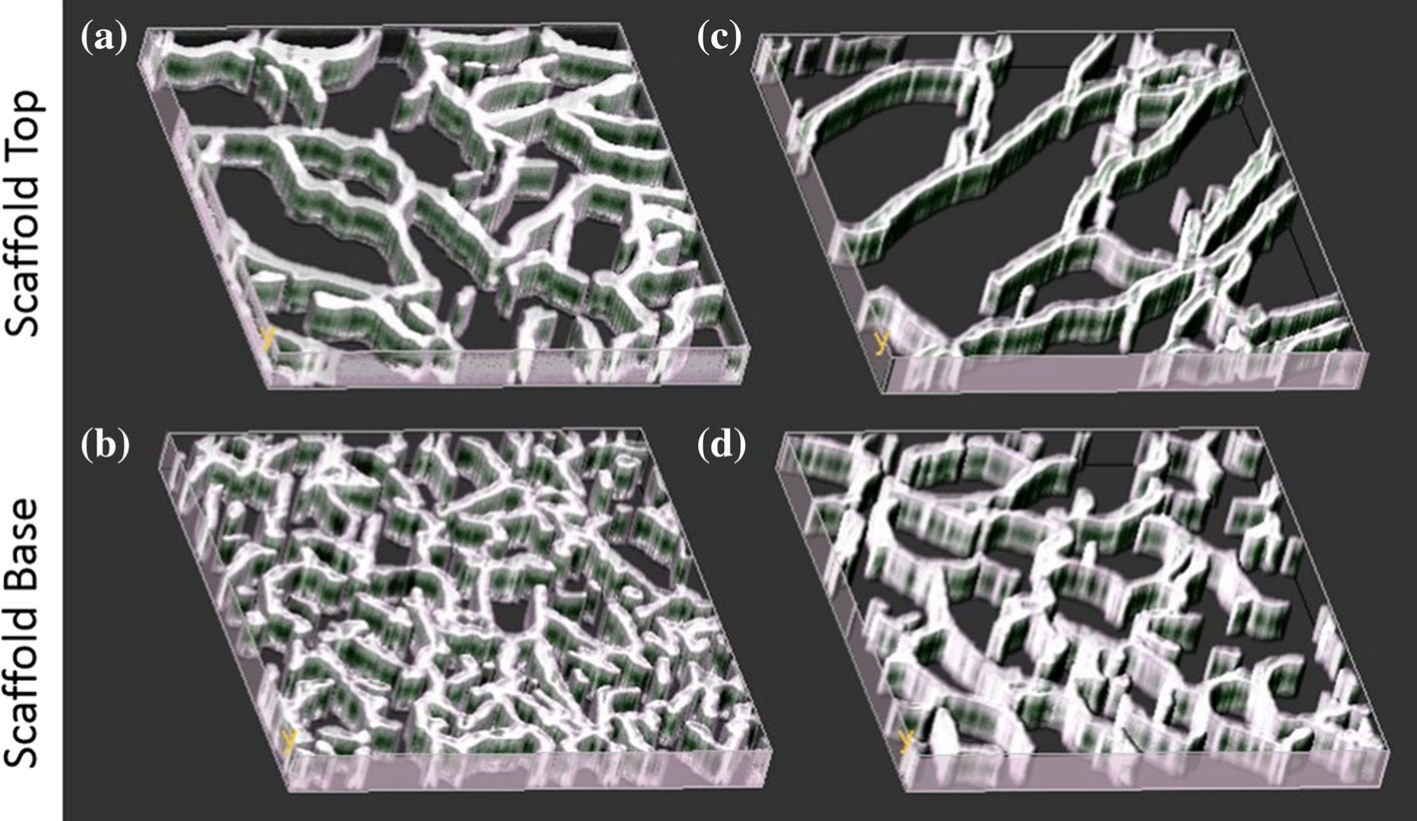
Scaffold structure changed as the freezing time for slurry increased from (**a**, ** b**) 1.5 h and (**c**, ** d**) 20 h in the perspex-36 % mold at both the ** a**, ** c** scaffold top and ** b**, ** d** scaffold base. All sections are 1 × 1 mm µCT reconstructions

Due to the insulating properties of the perspex molds, the temperature of the slurry during solidification was not equal to the set temperature of the freeze drier shelf, which acted as the heat sink. While the heat sink was set at −30 °C, the temperature within the slurry ranged from −18 to −13 °C from the base to the top. When a scaffold of large volume was treated with a lengthened freezing time of 20 h at −30 °C, compared to the usual 1.5 h, significant changes were observed in the architecture. The pore size at the base of the scaffold was significantly increased from 94 ± 5 to 112 ± 6 µm, Fig. 5. At the top, the structure of the pores not only increased in size, but started to show anisotropy as well.

## Discussion

Ice-templating techniques, such as freeze drying, are inherently dependent on thermal processes to create a well-defined structure. Control over structure, both the isotropy and the size of the scaffold features, can have a large impact on scaffold function. Within tissue engineering, isotropy is known to alter the matrix expression of musculoskeletal tissues, and features such as pore size are critical for the diffusion of nutrients and cells throughout the porous structures [5, 15]. With an understanding of the fundamental principles of ice growth, better solutions can be implemented to tailor the porous structures created via ice-templating.

### Collagen slurry solidification

It is important to note that cooling, prior to nucleation, and freezing, following nucleation, are two distinct processes. While cooling is a matter of heat conduction, the process of freezing is much more complex. As ice forms, the release of latent heat makes the slurry an additional heat source in the system. Thus, while the cooling of the slurry was not impacted by the contact with the heat sink in this study, during freezing the mold design was of critical importance.

The path of heat flow at the contact with the cold shelf during solidification was altered during freezing. This may be due to a change in the conductivity within the air gap with temperature and pressure changes. As a consequence, the contact area with the heat sink became the main thermal link in the system after nucleation of ice. As freezing time is highly dependent on the conduction of latent heat out of a slurry during solidification, it was significantly affected by the contact with the heat sink, and also by the filling volume which alters the amount of latent heat produced [9, 16]. Regardless of the filling volume, the mold with the largest air gap, perspex-80 %, had the longest freezing time, reaching of 70 min with a large filling volume.

### Latent heat release affects scaffold pore structure

By varying the key thermal link with the heat sink during freezing, in this case the contact area of the mold with the freeze drier shelf, the scaffold pore size was significantly affected. The mold with the largest air gap, perspex-80 %, produced scaffolds with the largest mean pore size distribution ranging from 95 ± 6 μm at the base to 183 ± 5 μm at the top. By severely restricting the ability of the heat to leave the slurry, the temperature remained near the equilibrium freezing temperature much longer at the slurry top. The longer time at equilibrium resulted in greater annealing of the ice structure, and thus, the largest pore sizes. As the shelf contact increased, and the thermal link between the slurry and the heat sink was improved, the pore size distribution decreased. This phenomenon has been demonstrated with materials other than perspex. Shelf contact was found to be critical in stainless steel molds for producing homogeneous scaffolds; areas with poor shelf contact had a larger pore size on average [17].

The effect of latent heat release was less visible with small filling volumes. The reduced volume of the slurry made heat removal more efficient. The reduction in the time at equilibrium also correlated to a reduction in pore size. At the base of the scaffolds, the pore size was constant across all scaffold types and filling volumes, remaining roughly 95 μm.

### Low-temperature ice annealing

While ice remodeling is most active around the equilibrium temperature, in the range of 0 to −1.5 °C, ice is still active at lower temperatures, although the rate of growth and annealing are significantly slower [13]. Consideration of low-temperature ice annealing is relevant when considering long-term holds, over 20 h, at low temperatures, below −20 °C, prior to drying, such as those associated with storage. With moderate contact with the heat sink, the internal temperatures of the slurry were significantly lower than the equilibrium temperature range, and yet there was a quantifiable change in pore structure. At the top of the scaffold, the ice crystals began to assume an anisotropic appearance due to the temperature gradient induced by the localized heat sink [18]. The phenomenon of anisotropy induced by holds at low temperature has also been reported in chitosan systems [19].

### Controlling ice growth during freezing

Collagen scaffold properties can be tuned with scaffold composition and cross-linking and have already demonstrated success in the regeneration of tendon, skin, and nerve [2, 15, 20–22]. While in vitro work and small animal models require scaffolds of relatively small dimensions, to expand the therapeutic potential of tissue engineering scaffolds, it will be increasingly necessary to adapt current scaffold technologies to create well-defined scaffold structures over large critical-sized defects, which can be over 10 mm in all dimensions [23].

To meet these challenges using ice-templating techniques, it is important to recognize that different processes of heat removal are in effect before and after ice nucleation. Both stages of freezing can be altered independently, as demonstrated in the current study. As the size of the scaffold increases, the distribution in pore size will increase unless contact with the heat sink is simultaneously improved to ensure the efficient flow of latent heat out of the slurry. To limit pore size distributions, low-temperature thermal holds should also be avoided, as they can also contribute to ice remodeling, even though temperatures are well below the equilibrium freezing temperature of ice.

## Conclusions

The solidification process which forms the structure of ice-templated scaffolds consists of two phases which can be controlled independently. During the freezing phase, which begins after ice nucleates in the slurry, contact with the heat sink, in this case the freeze drier shelf, was critical for controlling the path of heat removal from the slurry. Increasing contact with the heat sink significantly decreased the mean pore size at the top of the scaffold structures. The effects of shelf contact were less severe as the filling volume decreased. In addition, it was found that low-temperature thermal holds can also change the pore structure, by allowing further annealing of the ice crystals and leading to larger pore sizes and anisotropic pore structures. This insight into the freezing phase of solidification should influence future design and optimization of ice-templating techniques.

## References

[1] Deville S (2008). Freeze-casting of porous ceramics: a review of current achievements and issues. Adv Eng Mater.

[2] Yannas IV, Tzeranis DS, Harley BA, So PTC (2010). Biologically active collagen-based scaffolds: advances in processing and characterization. Philos Trans R Soc A.

[3] Yoshida S, Kimura Y, Ogino I, Mukai SR (2013). Synthesis of a microhoneycomb-type silica-supported ammonium molybdophosphate for cesium separation. J Chem Eng Jpn.

[4] O’Brien FJ, Harley BA, Yannas IV, Gibson LJ (2005). The effect of pore size on cell adhesion in collagen-gag scaffolds. Biomaterials.

[5] Murphy CM, Haugh MG, O’Brien FJ (2010). The effect of mean pore size on cell attachment, proliferation and migration in collagen-glycosaminoglycan scaffolds for bone tissue engineering. Biomaterials.

[6] Mortera-Blanco T, Mantalaris A, Bismarck A, Aqel N, Panoskaltsis N (2011). Longterm cytokine-free expansion of cord blood mononuclear cells in three-dimensional scaffolds. Biomaterials.

[7] Mullen LM, Best SM, Brooks RA, Ghose S, Gwynne JH, Wardale J, Rushton N, Cameron RE (2010). Binding and release characteristics of insulin-like growth factor-1 from a collagen-glycosaminoglycan scaffold. Tissue Eng Part C Methods.

[8] Campbell JJ, Davidenko N, Caffarel MM, Cameron RE, Watson CJ (2011). A multifunctional 3D co-culture system for studies of mammary tissue morphogenesis and stem cell biology. PLoS One.

[9] Pawelec KM, Husmann A, Best SM, Cameron RE (2014). Understanding anisotropy and architecture in ice-templated biopolymer scaffolds. Mater Sci Eng C.

[10] Husmann A, Pawelec KM, Burdett C, Best SM, Cameron RE (2015). Numerical simulations to determine the influence of mould design on ice-templated scaffold structures. J Biomed Eng Inf.

[11] Pawelec KM, Husmann A, Best SM, Cameron RE (2014). A design protocol for tailoring ice-templated scaffold structure. J R Soc Interface.

[12] Wilson C (1982). Texture and grain growth during the annealing of ice. Textures Microstruct.

[13] Hagiwara T, Mao J, Suzuki T, Takai R (2005). Ice recrystallization in sucrose solutions stored in a temperature range of −21 degrees C to −50 degrees C. Food Sci Technol Res.

[14] Pontius JS (1998). Estimation of the mean in line intercept sampling. Environ Ecol Stat.

[15] Pawelec KM, Wardale RJ, Best SM, Cameron RE (2015). The effects of scaffold architecture and fibrin gel addition on tendon cell phenotype.

[16] Tang XL, Pikal MJ (2004). Design of freeze-drying processes for pharmaceuticals: practical advice. Pharm Res.

[17] O’Brien FJ, Harley BA, Yannas IV, Gibson LJ (2004). Influence of freezing rate on pore structure in freeze-dried collagen-gag scaffolds. Biomaterials.

[18] Deville S, Maire E, Lasalle A, Bogner A, Gauthier C, Leloup J, Guizard C (2009). In situ x-ray radiography and tomography observations of the solidication of aqueous alumina particle suspensions- part i: initial instants. J Am Ceram Soc.

[19] Cooney MJ, Lau C, Windmeisser M, Liaw BY, Klotzbach T, Minteer SD (2008). Design of chitosan gel pore structure: towards enzyme catalyzed flow-through electrodes. J Mater Chem.

[20] Kew SJ, Gwynne JH, Enea D, Brookes R, Rushton N, Best SM, Cameron RE (2012). Synthetic collagen fascicles for the regeneration of tendon tissue. Acta Biomater.

[21] Grover CN, Cameron RE, Best SM (2012). Investigating the morphological, mechanical and degradation properties of scaffolds comprising collagen, gelatin and elastin for use in soft tissue engineering. J Mech Behav Biomed Mater.

[22] Grover CN, Gwynne JH, Pugh N, Hamaia S, Farndale RW, Best SM, Cameron RE (2012). Crosslinking and composition influence the surface properties, mechanical stiffness and cell reactivity of collagen-based films. Acta Biomater.

[23] Nuss KMR, Auer JA, Boos A, von Rechenberg B (2006). An animal model in sheep for biocompatibility testing of biomaterials in cancellous bones. BMC Musculoskelet Disord.

